# Urinary N-terminal titin fragment concentration as a non-invasive biomarker of exercise-induced muscle damage in males and females

**DOI:** 10.1007/s00421-025-05936-6

**Published:** 2025-08-12

**Authors:** Emily J. Hansell, Kirsty M. Reynolds, Jakob Škarabot, Lewis J. James, Tom Clifford, Josh Thorley

**Affiliations:** 1 School of Sport, Exercise and Health Sciences, Loughborough University, Loughborough, UK; 2 School of Medicine, University of Nottingham, Nottingham, UK; 3 NIHR Leicester Biomedical Research Centre, University Hospitals of Leicester NHS Trust and University of Leicester, Leicester, UK

**Keywords:** Muscle damage, Titin, Creatine kinase, Exercise, Biomarker

## Abstract

**Purpose:**

To examine the effects of muscle-damaging exercise on urinary N-terminal fragments of titin (UTF) in males and females, and its association with markers of exercise-induced muscle damage.

**Methods:**

27 males (*n* = 16) and females (*n* = 11) (height: 1.74 ± 0.10 m; body mass: 72.2 ± 11.4 kg; age: 22 ± 3 years) performed 200 eccentric contractions of the knee extensor on an isokinetic dynamometer. Urine and serum samples were collected pre-, post- and 48 h post-exercise to quantify UTF and creatine kinase (CK). Additionally, knee extensor maximal voluntary isometric force (MVIF), voluntary activation (VA), time to peak twitch (TTP), evoked maximal rate of force development (RFD_max_), potentiated twitch force (Tw_pot_), and delayed-onset muscle soreness (DOMS) were recorded.

**Results:**

UTF (2.3 ± 1.8 to 3.3 ± 3.4 nmol/mg/dL) and CK (9.7 ± 4.8 to 14.5 ± 8.7 units/L) concentrations were elevated 48 h after exercise (*p* < 0.01). DOMS was greater at all post-exercise time points vs. pre-exercise (*p* < 0.01). MVIF, evoked RFD_max_, VA, and Tw_pot_ all decreased after exercise (*p* < 0.01). The pre- to 48 h post-exercise change in UTF strongly correlated with CK (r_s_ = 0.73; *p* < 0.01), TTP (r_s_ = -0.77; *p* < 0.01) and evoked RFD_max_ (r_s_ = -0.62; *p* < 0.01) and moderately correlated with MVIF (r_s_ = -0.45; *p* < 0.01). Moderate strength correlations were found between the pre- to 48 h post-change in CK with DOMS (r = 0.47; *p* = 0.03). There were no sex differences for any variables (*p* > 0.05).

**Conclusion:**

UTF was similarly increased post- and 48 h post-exercise in males and females and was moderately to strongly correlated to CK and some markers of neuromuscular function, but not DOMS.

**Supplementary Information:**

The online version contains supplementary material available at 10.1007/s00421-025-05936-6.

## Introduction

Exercise-induced muscle damage (EIMD) is loosely defined as a set of symptoms typically evoked by strenuous, unaccustomed, and eccentric-heavy exercise. While EIMD can be measured directly by examining myofibrillar disruptions from muscle biopsy (Friden et al. 1983; Fridén and Lieber 2001), obtaining muscle samples is highly invasive. As such, EIMD is often measured indirectly as declines in muscle force production and an increase in intramuscular proteins in the blood, where changes often peak 24–72 h post-exercise (Howatson and Van Someren 2008). Several of these intramuscular proteins, most notably creatine kinase (CK), are significantly elevated following muscle-damaging exercise (Hyldahl and Hubal 2014). As such, CK has been widely adopted as a biomarker of EIMD, especially in the absence of neuromuscular function testing (Warren et al. 1999; Brancaccio et al. 2010). However, the use of CK as a marker of EIMD has been questioned, since it is influenced by several other factors, including biological sex, hydration status, training status, and fibre type distribution (Nosaka and Clarkson 1996; Seifert et al. 2005; Brancaccio et al. 2007). Moreover, CK, and other proteins such as myoglobin, do not correlate with myofibrillar damage or declines in muscle force production (Margaritis et al. 1999; Fridén and Lieber 2001), the latter of which is considered the gold standard marker of EIMD (Warren et al. 1999). Collection of these intramuscular proteins also requires invasive procedures such as venepuncture, making them difficult to collect in field settings or in participants with a fear of needles. Hence, there is growing interest in the effects of exercise on other intramuscular proteins that could be less invasive and better reflect the magnitude of EIMD.

One such protein is titin (also known as connectin) which is a giant protein located in the sarcomere of striated muscle that enables the contraction of actin-containing thin filaments and myosin-containing thick filaments. Thus, titin serves as a molecular spring that provides elasticity and passive stiffness to myofibers (Herzog 2018). When muscle is damaged, titin is cleaved by proteolytic calpain and matrix metalloprotease enzymes, forming N-terminal titin fragments that are excreted in urine (Ali et al. 2010; Charton et al. 2015). Following the development of a highly sensitive enzyme-linked immunosorbent assay (ELISA) to quantitatively measure the concentration of urinary titin fragments (UTF) (Maruyama et al. 2016), recent studies have shown that UTF is elevated after muscle-damaging exercise (Kanda et al. 2017; Yamaguchi et al. 2020c, b; Tanabe et al. 2021a, b), and in clinical populations with muscle atrophy (Matsuo et al. 2019; Nakanishi et al. 2021).

Kanda et al. (2017) were the first to report that strenuous exercise (100 eccentric-biased calf raises) increased UTF concentrations in males, leading the authors to recommend UTF as a potentially useful biomarker to detect EIMD. More recently, Yamaguchi et al. (2020c) demonstrated elevated UTF levels in males after 30 maximal isokinetic eccentric contractions of the elbow flexor and reported moderate negative correlations between peak UTF levels and decreases in neuromuscular function (peak maximal voluntary isometric force (MVIF)) 24–144 h post-exercise. Tanabe et al. (Tanabe et al. 2021b) similarly reported elevated UTF concentrations 48 h following 30 maximal isokinetic eccentric contractions of the elbow flexor, with peak values strongly positively correlated with serum CK activity and transverse relaxation time. Furthermore, there were moderate negative correlations between peak UTF and MVIF and range of motion decline rate. Another study by Tanabe et al. (2021a) also found that a 90 min soccer match in male collegiate players elevated UTF 48 h post-exercise, with concentrations moderately negatively correlated with a decline in countermovement jump (CMJ) performance.

While previous studies suggest that UTF concentrations may serve as a non-invasive biomarker for detecting EIMD, current evidence is limited by small sample sizes (*n* = 8–17), comparison to a narrow range of neuromuscular assessments (Kanda et al. 2017; Yamaguchi et al. 2020c, Yamaguchi et al. 2020b; Tanabe et al. 2021a), and a lack of sex-based comparisons—despite indications that EIMD may be attenuated in females compared to males (Kendall and Eston 2002; Penailillo et al. 2011). Importantly, no study to date has examined the relationship between UTF and laboratory-based markers of EIMD such as evoked force and voluntary activation (VA). Accurate interpretation of maximal force loss following exercise requires consideration of central drive, yet central activation failure is rarely accounted for in EIMD research (Warren et al. 1999). As reductions in force could stem from impaired voluntary neural input rather than structural muscle damage alone, the use of twitch interpolation is recommended to objectively distinguish between central and peripheral contributions to muscle function loss (Byrne et al. 2004; Millet and Lepers 2004). Accordingly, we have incorporated this technique to provide a more comprehensive assessment of the neuromuscular response to EIMD and to better evaluate the sensitivity of UTF as a biomarker.

The primary aim of this study was therefore to determine whether UTF is increased by muscle-damaging exercise and whether the response differs between males and females. A secondary aim was to correlate changes in UTF with changes to an array of neuromuscular function markers and muscle soreness. We also measured CK and correlated exercise-induced changes with neuromuscular function and muscle soreness for comparisons to UTF correlations. We hypothesised that muscle-damaging exercise would increase UTF and CK, but to a lesser extent in females, and that these changes would correlate with decrements in neuromuscular function and muscle soreness after 48 h, albeit more strongly with UTF than CK.

## Methods

### Ethical approval

The study received ethical approval from the Loughborough University Ethics Approvals (human participants) Sub-Committee (REF: 4955). Participants were fully informed of the risks and discomforts associated with the experimental procedures prior to providing written informed consent.

### Participants

27 participants (height: 1.74 ± 0.10 m; body mass: 72.2 ± 11.4 kg; age: 22 ± 3 years; see Table 1 for physical characteristics), including males (*n* = 16) and females (*n* = 11), volunteered to participate in this study. All participants were recreationally strength trained (defined as undertaking resistance training 1–4 days per week for the past 6 months) and deemed healthy following examination of a health screening questionnaire; this meant participants (1) had not experienced any muscle injuries in the past 6 months (2) were not using medication, (3) had a BMI between 18.5 and 30.0 kg/m^2^, (4) and had no known underlying health conditions. Female participants were only eligible for inclusion if they were either naturally menstruating or using a combined oral contraceptive pill. Trials were completed during the early follicular phase of the menstrual cycle or the first week of the active pill cycle. The data presented in this manuscript formed part of a larger study examining the effects of a dietary intervention on EIMD. As there were no significant differences in the outcomes between the two interventions, data from both groups was used for analysis.

**Table 1:** Group differences in physical characteristics and total work completed during exercise

	Males (*n* = 16)	Females (*n* = 11)	*p*
Age (years)	23 ± 3	23 ± 4	0.82
Height (m)	1.81 ± 0.05	1.64 ± 0.07	< 0.01
Body mass (kg)	78.4 ± 9.9	63.0 ± 5.9	< 0.01
Total work (J/kg BM)	549 ± 85	458 ± 128	0.04
MVIF (N/kg BM)	10.6 ± 1.8	9.3 ± 1.7	0.07

MVIF, maximal voluntary isometric force per kg of body mass (BM) at baseline

### Study design

Data were collected across three laboratory visits: a familiarisation trial, an exercise trial, and a 48 h follow-up trial. During familiarisation, anthropometric measurements were collected, and participants were familiarised with the muscle soreness and neuromuscular function tests; for the latter, they experienced peripheral femoral nerve stimulation. Due to the discomfort of nerve stimulation, participants were given the opportunity to opt out of this aspect for subsequent visits. On the day of the exercise, data was collected pre- and immediately post-exercise. Participants returned to the laboratory 48 h post-exercise. On both visits, participants arrived at the laboratory in the morning following an overnight fast and were hydrated following consumption of 500 mL water. The 48 h post-exercise time point was chosen for follow-up because systemic changes in myofiber proteins, and urinary titin, do not tend to increase significantly until this time point (Warren et al. 1999; Clarkson and Hubal 2002; Kanda et al. 2017; Yamaguchi et al. 2020a). Participants were instructed to avoid exercise outside of study procedures 36 h prior to all trials. Nutritional supplements and therapeutic interventions aimed to enhance recovery (e.g. compression garments, cooling devices) were also restricted from 7 days prior to exercise until completion of the study.

### Muscle-damaging exercise protocol

To induce muscle damage, participants performed 200 dominant-leg eccentric knee flexions on an isokinetic dynamometer (IKD) (HUMAC NORM, CSMi, Stoughton (MA), United States). Participants were seated at a hip angle of 125° and strapped securely across the chest and inguinal fold. The IKD range of motion limits were set individually with a total range of motion of ~ 90°. Contractions were performed in 20 sets of 10, with 1 min between-set rest, and at a constant angular velocity of 60°·s^−1^. Participants initiated the contraction by ‘kicking’ from a 10° joint angle and subsequently resisting the action of the IKD lever arm as it moved to the participants’ flexed position. During the concentric phase of action, participants were instructed to relax as the lever arm returned their leg to the start position. Participants were encouraged to perform each contraction maximally by the same investigator, and visual feedback was provided via a linked PC. Total work completed (J) was recorded and used for analysis.

### Neuromuscular function

Neuromuscular function was evaluated using knee flexion dynamometry on the dominant leg; this was performed on a custom-built isometric strength testing chair tailored to each participant's anthropometric measurements. Participants were seated with the knee and hip positioned at 115° and 125° of extension, respectively. Movement of the upper body and hips was restricted using adjustable straps secured across the torso and hips. The dominant leg's shin was fastened with a cuff and reinforced canvas webbing above the lateral malleolus, at approximately 15% of the tibial length. The cuff, aligned perpendicular to the tibia, was connected in series with a calibrated S-beam strain gauge (Force Logic, Swallowfield, UK). After signal amplification (× 370), force data was sampled and recorded at 2000 Hz using an external analogue–digital converter (Micro 1401; CED Ltd., Cambridge, UK) and Spike2 PC software (CED Ltd., Cambridge, UK).

Participants performed a standardised warm-up consisting of ~ 3 s submaximal isometric knee extensions at 50% (× 3), 75% (× 3) and 90% (× 1) of perceived maximal effort, with each contraction separated by 30 secs of rest. After the warm-up, participants rested for 2 min, then performed 5 sets of ∼3 s MIVCs separated by 30 secs of rest to assess MVIF.

15 participants (male: *n* = 9; female: *n* = 6) consented to undertake peripheral femoral nerve stimulation. Prior to being seated in the testing chair, two self-adhesive electrodes (PALS879100, Axelgaard, Fallbrook (CA), USA) were attached to the participant's’ dominant leg (anode: greater trochanter of the femur; cathode: over the femoral triangle/intersection of adductor longus, inguinal ligament and sartorius). Electrical stimulus intensity was standardised across participants via calibration on each test occasion prior to the neuromuscular warm-up. Stimulation was initially delivered at 20 mA, before being increased in 20mA stepwise increments until a plateau in knee extensor evoked twitch was obtained. Stimulus intensity for maximal voluntary isometric contraction (MVIC) efforts was set at 130% of this value to ensure supramaximal delivery.

During the final three MVICs, percutaneous single and paired electrical stimuli were delivered to the femoral nerve using square wave pulses (200 μs) via a constant current stimulator (DS7AH; Digitimer Ltd., Welwyn Garden City, UK). Pulses were delivered superimposed at the peak of maximal contraction (one paired pulse at 100 Hz) and post-contraction at rest interspersed by ~ 2–3 s (one 100 Hz paired pulse, one 10 Hz paired pulse, and a single pulse). Voluntary activation (VA) was calculated using the interpolated twitch technique (VA (%) = (1− (superimposed twitch/100 Hz paired potentiated twitch) × 100)). Evoked maximal rate of force development (RFD_max_) was calculated from the single pulse potentiated twitch response—as the maximal slope of the force response calculated in 100 ms epochs. Potentiated twitch force (Tw_pot_) and time to peak twitch (TTP) were determined using the 100 Hz single pulse.

These neuromuscular markers were chosen since most appear to be sensitive to muscle-damaging exercise and would therefore help to validate whether changes to UTF reflected EIMD. MVIF can be seen to be blunted for up to 48 h post-exercise (Prasartwuth et al. 2005; Turner et al. 2008; Power et al. 2010; Behrens et al. 2012; Doguet et al. 2016). VA was shown to decrease immediately post-exercise (Prasartwuth et al. 2005; Doguet et al. 2016). RFD_max_ was reported to drop immediately post-exercise, staying suppressed for up to 168 h (Molina and Denadai 2012; Jenkins et al. 2014; Farup et al. 2016; Vila-Chã et al. 2023). Janecki et al. (2014) reported that Tw_pot_ was diminished immediately post-muscle-damaging exercise and remained attenuated for up to 120 h. To our knowledge, no present study has examined TTP response to muscle-damaging exercise and thus it was of interest to evaluate these responses for the first time.

### Muscle soreness

Delayed-onset muscle soreness (DOMS) was evaluated using a visual analogue scale. Participants performed a body weight squat to a 90° knee angle and were then asked to mark a point on a 200 mm line, where 0 mm indicated ‘no soreness’ and 200 mm indicated ‘unbearably painful’. The mark was subjectively placed at the spot that best represented their perceived soreness in the lower limbs. The distance of the mark from the start of the line was measured in mm and recorded.

### Sample collection and analysis

Spot urine and venous blood samples were collected at all time points; venous blood was collected from the antecubital fossa via venepuncture by a trained phlebotomist and was left to clot for 30 min at room temperature, followed by centrifugation at 3500 RCF for 10 min at 4°C to isolate serum. Urine and serum samples were then frozen at − 80°C until analysed.

UTF was analysed using a commercial sandwich ELISA (Titin N-terminal Fragment Assay Kit; Immuno-Biological Laboratories Co., Fujioka, Japan) in duplicate according to manufacturer’s instructions. Briefly, urine samples were thawed and diluted between 1:20 and 1:50, then added to an antibody-coated 96-well plate where they underwent incubation at 37 °C for 1 h. After repeating a wash procedure four times, labelled antibodies were added to the plate then incubated at 37°C for 30 min. Following another five washes, tetramethylbenzidine substrate was added to wells and the plate was incubated at room temperature away from light exposure for 30 min. Upon colour development, the reaction was terminated by the addition of stop solution, and the absorbance of wells was read at 450 nm with a reference wavelength of 650 nm using a microplate reader. To account for transient changes in hydration across the time points, UTF (nmol/L) was normalised to creatinine concentrations (mg/dL) which were performed using a commercial assay (Creatinine Urinary Colorimetric Assay Kit, Cayman Chemicals, Ann Arbor, USA) in duplicate according to the manufacturer’s instructions. UTF is therefore expressed as nmol/mg/dL. The intra-assay CV for the UTF and creatinine assays were 3.2% and 3.7%, respectively.

Serum CK was analysed using a commercial enzyme activity assay (CK Activity Assay Kit; Sigma-Aldrich, St. Louis, MA, USA) according to the manufacturer’s instructions. CK activity in serum (units/L) was quantified by a coupled enzyme reaction resulting in the formation of NADPH that was measured at 340 nm. The formation of NADPH was subsequently proportionate to CK concentration, and 1 unit of CK is the amount of enzyme required to transfer 1 µM of phosphate from phosphocreatine to ADP per minute.

### Statistical analysis

Statistical analysis was performed using jamovi v2.3.26 (retrieved from https://www.jamovi.org). Normal distribution of data was confirmed with the Shapiro–Wilk test (*p* > 0.05) and by inspecting the Q–Q plots and histograms of the residuals. Independent samples t-tests were used to analyse biological sex differences in physical characteristics and total work performed during exercise. Linear mixed models (LMM; gamlj v2.6.6) (2 (males vs. females) × 3 (pre-, post-, 48 h post-exercise)) were performed on the UTF, CK, DOMS, and neuromuscular responses to exercise. Prior to LMM analysis, raw values were converted to per cent (%) change from pre-exercise to account for the anthropometric and physiological differences between male and female participants. Any significant main effects from the LMM were followed up with Holm–Bonferroni corrected post hoc tests. Effect sizes for LMM analysis are presented as partial eta squared (ηp^2^: small: 0.01, medium: 0.06, large 0.14) (Cohen 1988).

Since UTF concentrations were not normally distributed, Spearman’s ranked correlations were performed to examine associations between the change (from pre- to 48 h post-exercise) in UTF with the change in CK, DOMS, and neuromuscular function. Pearson’s correlations were used to examine associations between CK, DOMS, and neuromuscular function. As in a previous study (Kanda et al. 2017), correlations were performed with % change data to standardise the changes between the two different outcomes (e.g. UTF and DOMS). Correlation analysis was not performed for pre-post changes, as UTF would not be expected to significantly increase immediately post-exercise as a result of muscle damage. Spearman’s (r_s_) and Pearson’s (r) correlation coefficients were interpreted as follows: ± 0.1 to ± 0.3 *weak;* ± 0.4 to ± 0.6 *moderate;* ± 0.7 to ± 0.9 *strong* (Dancey and Reidy 2007). *p* < 0.05 was considered statistically significant. Figures were generated using GraphPad Prism (v9.4.1, Boston, USA). Data is presented as mean ± SD.

## Results

Physical characteristics, total work completed during exercise, and MVIF at baseline between males and females are detailed in Table 1.

### Changes in urinary titin fragments after exercise

**Fig. 1:**
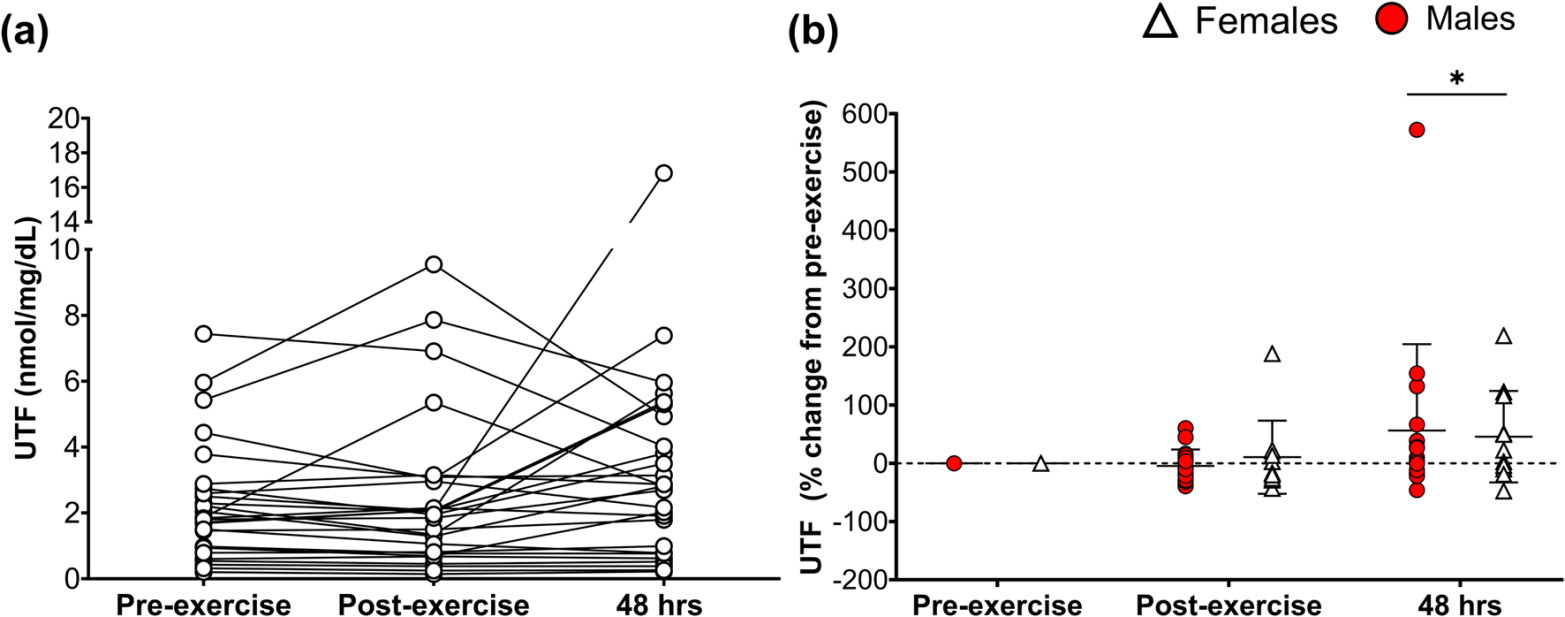
Urinary titin fragment (UTF) responses following muscle-damaging exercise in males (red circles) and females (white triangles). **a** Individual raw UTF concentrations in all participants (*n* = 27). **b** % Change (from pre-exercise) of UTF at post- and 48 h post-exercise between males (*n* = 16) and females (*n* = 11). **b** Presents individual data points alongside mean and SD. * = significantly different from pre- and post-exercise

Figure 1a presents the raw UTF concentrations after exercise for males and females. Figure 1b presents the % change in UTF after exercise for males and females. Main time effects were observed for UTF concentrations (time:* p* = 0.02; ηp^2^ = 0.11; Fig. 1b); UTF levels post-exercise were not different from pre-exercise (*p* = 0.88), but after 48 h, UTF was higher than pre-exercise (*p* < 0.01) and post-exercise (*p* = 0.02). No sex differences (*p* = 0.88; ηp^2^ < 0.01) or interaction effects (*p* = 0.71; ηp^2^ < 0.01) were found for UTF.

### Changes in serum creatine kinase after exercise

**Fig. 2:**
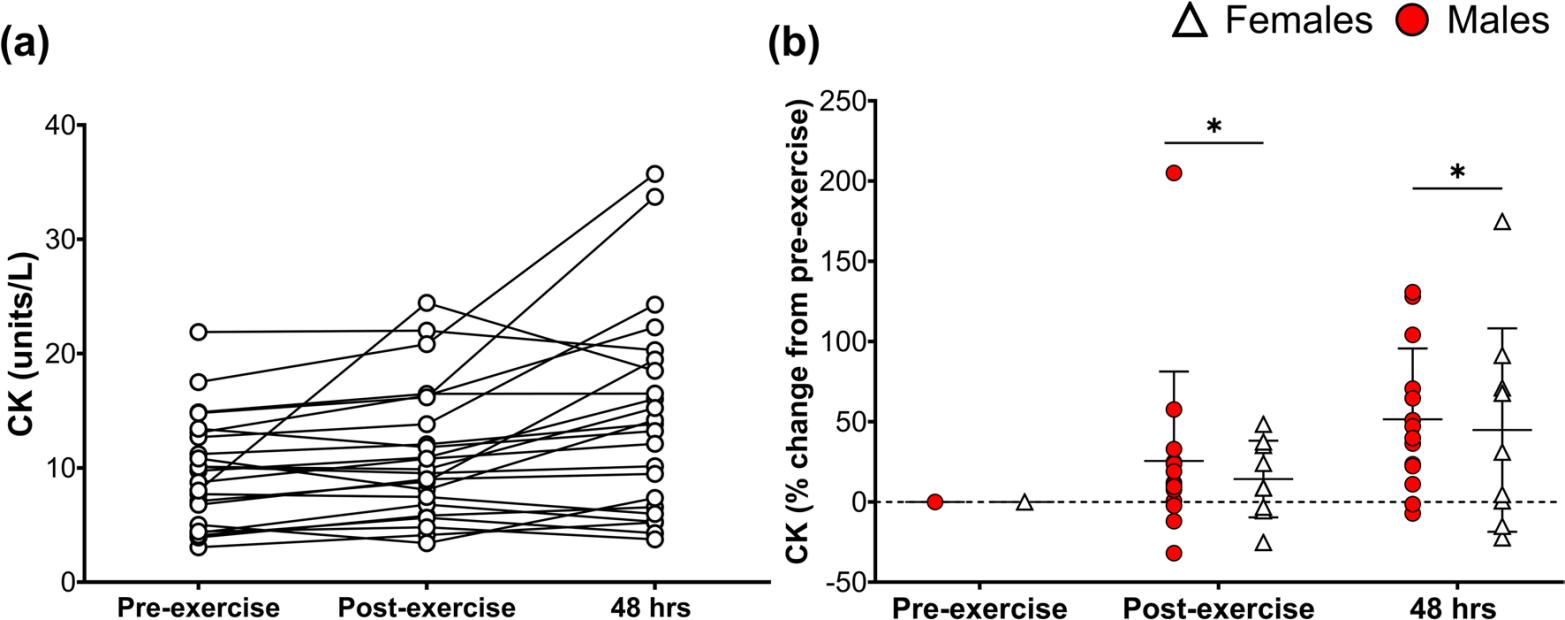
Creatine kinase (CK) responses following muscle-damaging exercise in males (red circles) and females (white triangles). **a** Individual raw CK concentrations in all participants (*n* = 23). **b** % Change (from pre-exercise) of UTF at post- and 48 h post-exercise between males (*n* = 14) and females (*n* = 9). **b** Presents individual data points alongside mean and SD. * = significantly different from pre-exercise

Serum samples could not be collected from *n* = 4; thus, CK was analysed for 23 participants. Figure 2a presents the raw CK concentrations after exercise for males and females. Figure 2b shows the % change of CK after exercise for males and females. Main time effects were found for CK (*p* < 0.01; ηp^2^ = 0.32; Fig. 2b); CK responses post-exercise and 48 h post-exercise were both elevated compared to pre-exercise, peaking 48 h post-exercise (*p* < 0.01) (Fig. 2b). No sex differences (*p* = 0.62; ηp^2^ = 0.02) or interaction effects (*p* = 0.87; ηp^2^ < 0.01) were observed for CK responses after exercise.

### Changes in muscle damage markers after exercise

**Fig. 3:**
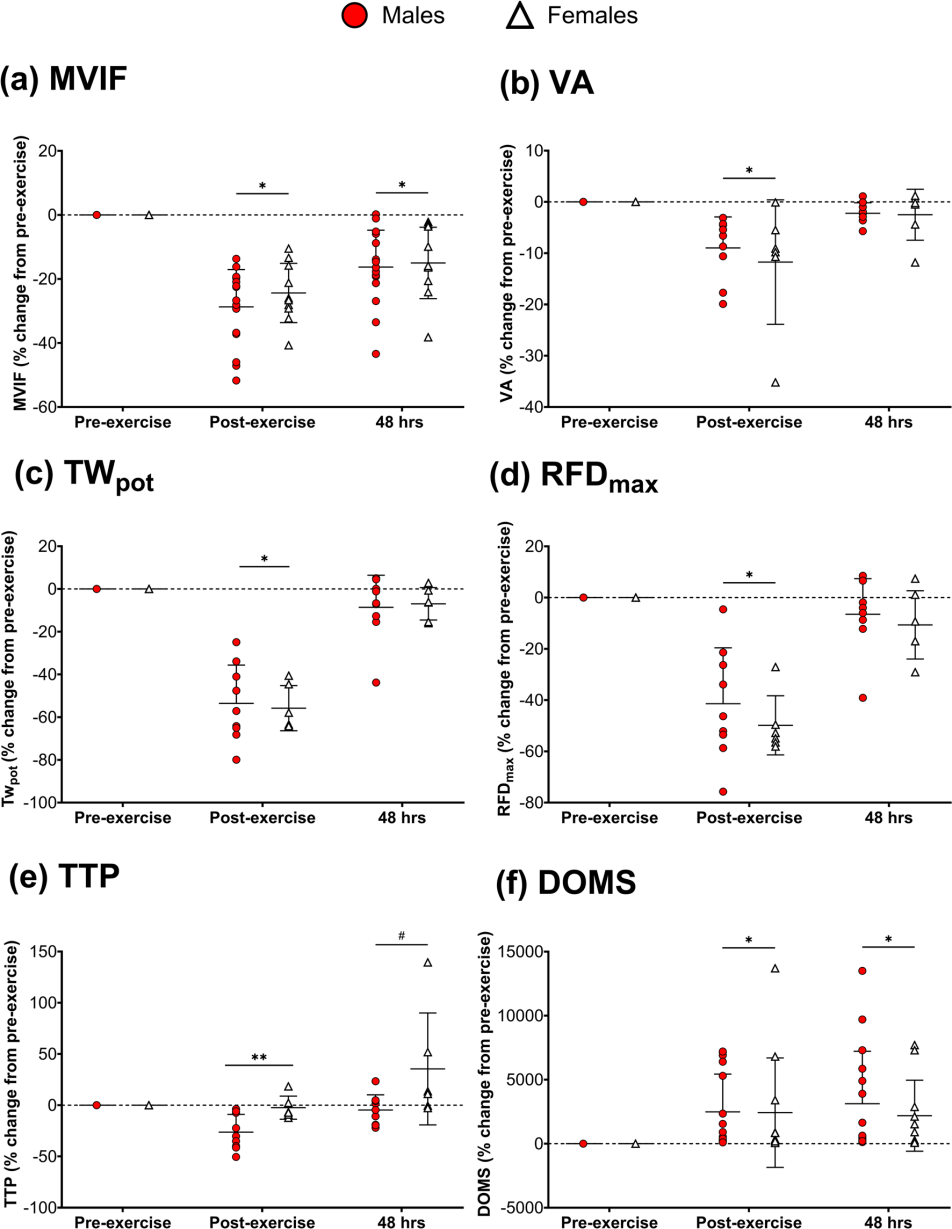
Changes in neuromuscular function and soreness markers after exercise in males and females. **a** Maximal voluntary isometric force (MVIF) (*n* = 27); **b** voluntary activation (VA) (*n* = 17); **c** potentiated twitch force (Tw_pot_) (*n* = 17); **d** evoked maximal rate of force development (RFD_max_) (*n* = 17); **e** time to peak twitch (TTP) (*n* = 17); **f** delayed-onset muscle soreness (DOMS) (*n* = 27). All figures are presented as individual data points alongside mean and SD. * = different from pre-exercise. ** = significantly different from 48 h post-exercise. # = interaction effect

Changes in muscle damage markers are presented here as % change from pre-exercise; raw values can be found in Table S1. There were main time effects for MVIF (*p* < 0.01; ηp^2^ = 0.77; Fig. 3a), VA (*p* < 0.01; ηp^2^ = 0.63; Fig. 3b), Tw_pot_ (*p* < 0.01; ηp^2^ = 0.93; Fig. 3c), evoked RFD_max_ (*p* < 0.01; ηp^2^ = 0.84; Fig. 3d), TTP (*p* < 0.01; ηp^2^ = 0.38; Fig. 3e) and DOMS (*p* < 0.01; ηp^2^ = 0.31; Fig. 3f).

MVIF decreased post-exercise (*p* < 0.01) and remained attenuated after 48 h post-exercise (*p* < 0.01). VA, Tw_pot_ and evoked RFD_max_ all decreased post-exercise (*p* < 0.01 for all), but returned to their respective baseline values 48 h post-exercise (VA: *p* = 0.58; Tw_pot_: *p* = 0.12; RFD_max_: *p* = 0.15). TTP did not change post-exercise (*p* = 0.20), but immediately post-exercise, TTP was faster vs. 48 h post-exercise (*p* < 0.01). DOMS increased post-exercise (*p* < 0.01) and remained elevated 48 h post-exercise (*p* < 0.01).

No sex differences or interaction effects were observed for MVIF (group: *p* = 0.19; ηp^2^ = 0.07; interaction: *p* = 0.30; ηp^2^ = 0.03), VA (group: *p* = 0.86; ηp^2^ < 0.01; interaction: *p* = 0.97; ηp^2^ < 0.01), Tw_pot_ (group: *p* = 0.98; ηp^2^ < 0.01; interaction: *p* = 0.80; ηp^2^ = 0.02), evoked RFD_max_ (group: *p* = 0.44; ηp^2^ = 0.05; interaction: *p* = 0.61; ηp^2^ = 0.04), and DOMS (group: *p* = 0.71; ηp^2^ < 0.01; interaction: *p* = 0.71; ηp^2^ = 0.01).

Main group (*p* = 0.02 ηp^2^ = 0.35) and interaction (*p* = 0.04; ηp^2^ = 0.22) effects were evident for TTP, as males demonstrated quicker TTP than females. At 48 h post-exercise, TTP had recovered closer to baseline in males compared to females (*p* = 0.03).

### Correlations between urinary titin fragments and muscle damage markers

**Fig. 4:**
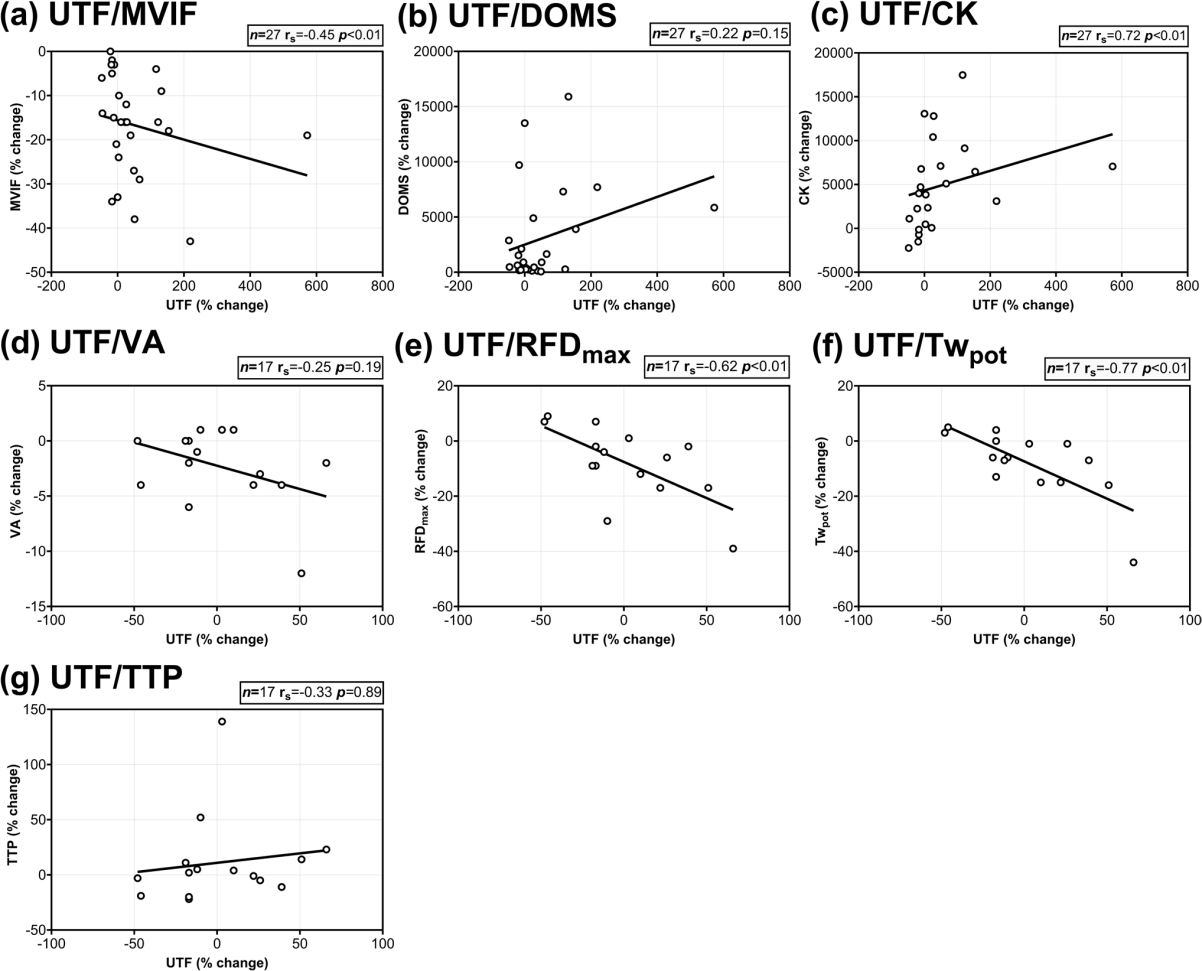
Spearman’s correlations coefficients (r_s_) between the per cent (%) change (from pre- to 48 h post-exercise) of urinary titin fragments (UTF) and **a** maximal voluntary isometric force (MVIF) (*n* = 27), **b** delayed-onset muscle soreness (DOMS) (*n* = 27), **c** creatine kinase (CK) (*n* = 27), **d** voluntary activation (VA) (*n* = 17), **e** evoked maximal rate of force development (RFD_max_) (*n* = 17), **f** potentiated twitch force (Tw_pot_) (*n* = 17), **g** time to peak twitch (*n* = 17)

Moderate negative correlations were observed between UTF and MVIF (r_s_ = −0.45; *p* < 0.01; Fig. 4a), whilst strong negative correlations were observed with Tw_pot_ (r_s_ = −0.77; *p* < 0.01; Fig. 4f) and evoked RFD_max_ (r_s_ = −0.62; *p* < 0.01; Fig. 4e). A strong positive correlation was found for UTF and CK (r_s_ = 0.72; *p* < 0.01; Fig. 4c). No significant correlations were observed between UTF and DOMS (r_s_ = 0.22; *p* = 0.15; Fig. 4b), VA (r_s_ = −0.25; *p* = 0.19; Fig. 4d) and TTP (r_s_ = 0.33; *p* = 0.89; Fig. 4g). Table S2 presents Spearman’s correlation coefficients between UTF and soreness and neuromuscular function markers in males and females. In both males and females, strong negative correlations were found between UTF and Tw_pot_.

### Correlations between serum creatine kinase and muscle damage markers

**Fig. 5:**
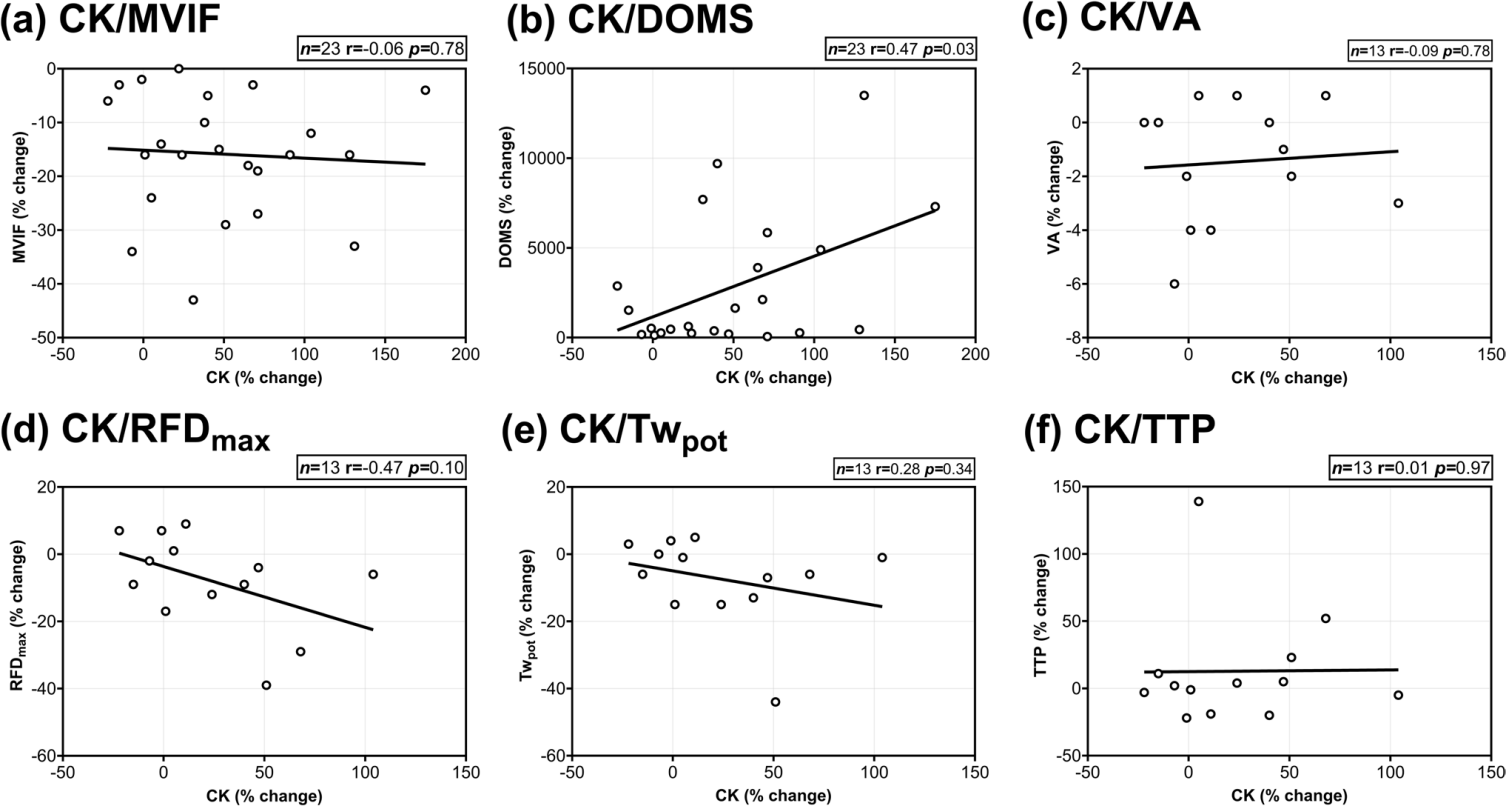
Pearson’s correlations coefficients (r) between the per cent (%) change (from pre- to 48 h) of creatine kinase (CK) and **a** maximal voluntary isometric force (MVIF) (*n* = 23); **b** delayed-onset muscle soreness (DOMS) (*n* = 23), **c** voluntary activation (VA) (*n* = 13), **d** evoked maximal rate of force development (RFD_max_) (*n* = 13), **e** potentiated twitch force (Tw_pot_) (*n* = 13), **f** time to peak twitch (TTP) (*n* = 13)

Moderate strength correlations were found between the % change in CK and DOMS (r = 0.47; *p* = 0.03; Fig. 5b) and evoked RFD_max_ (r = −0.47; *p* = 0.10; Fig. 5d). No significant correlations were found between CK and MVIF (r = −0.06; *p* = 0.78; Fig. 5a), VA (r = −0.09; *p* = 0.78; Fig. 5c), Tw_pot_ (r = 0.28; p = 0.34; Fig. 5e), and TTP (r = 0.01; *p* = 0.97; Fig. 5f).

## Discussion

This study explored whether (a) muscle-damaging exercise increased UTF in both males and females; (b) there were any sex differences in post-exercise UTF responses, and (c) whether changes in UTF levels 48 h after exercise correlated with various indirect markers of EIMD. The main findings indicate that UTF concentrations are similarly elevated 48 h after muscle-damaging exercise in both males and females, and increases in UTF are more strongly correlated with decrements in neuromuscular function than CK. In contrast, increases in CK are more closely correlated to DOMS, but there is a strong positive correlation between UTF and CK. These findings suggest that UTF may be a useful non-invasive, indirect, biomarker of myofibrillar disruption after strenuous eccentric exercise in males and females.

Several recent studies suggest that UTF may serve as a non-invasive biomarker for detecting EIMD in healthy individuals, as it reliably increases following damaging exercise and correlates with declines in muscle function and elevations in DOMS (Kanda et al. 2017; Yamaguchi et al. 2020c, Yamaguchi et al. 2020b; Tanabe et al. 2021a, Tanabe et al. 2021b). Notably, UTF concentrations rise more substantially following eccentric-biased exercise compared to concentric-biased protocols, reinforcing the idea that its elevation is specifically linked to mechanical muscle damage rather than general exercise-induced physiological stress (Yamaguchi et al. 2020a). Additionally, one study reported that UTF responses are attenuated following a repeated bout of exercise, suggesting that UTF may be influenced by training status and the repeated bout effect (Yamaguchi et al. 2020b). Collectively, these findings point to UTF as a potentially more sensitive and specific marker of EIMD than traditional biomarkers such as CK, whose validity has been increasingly questioned (Brancaccio et al. 2007). Furthermore, the clinical utility of UTF is supported by its use as a biomarker in muscle-related pathologies, including Duchenne muscular dystrophy and idiopathic inflammatory myopathies (Ishii et al. 2023; Sun et al. 2023), underscoring its relevance in conditions characterised by elevated muscle damage and catabolism.

In agreement with previous studies (Kanda et al. 2017; Yamaguchi et al. 2020c; Tanabe et al. 2021b), the present study demonstrates that high-volume, maximal eccentric contractions elevated UTF concentrations in some, but not all, participants 48 h post-exercise. Eccentric contractions are well known to induce structural damage to skeletal muscle structures (Cermak et al. 2012; Deyhle et al. 2016). Specifically, repeated overstretching during eccentric loading leads to morphological disruption of sarcomeres, including damage to Z-lines (Friden et al. 1983)—the anchoring regions where titin connects to the M-line and contributes to sarcomerogenesis and myofibrillar assembly (Prado et al. 2005). Direct evidence of titin disruption comes from Trappe et al. (2002), who reported an approximate 30% reduction in titin content in vastus lateralis biopsies following a single bout of high-intensity eccentric exercise. Although the precise mechanisms underlying titin breakdown remain inconclusive, it is widely theorised that focal sarcomeric damage is followed by an uncontrolled Ca^2^⁺ influx into the myofibrillar cytosol (Gissel 2005), which activates calcium-dependent proteolytic enzymes, such as calpains and matrix metalloproteinases, leading to the degradation of key structural proteins, including titin (Beckmann and Spencer 2008; Ali et al. 2010). The activation of calpains, alongside the release of pro-inflammatory mediators, further compromises sarcolemmal integrity and sarcomeric stability (Letavernier et al. 2012; Fatouros and Jamurtas 2016). It is therefore plausible that the rise in UTF observed here and in other studies reflects the proteolytic degradation of titin, combined with increased sarcolemma permeability, enabling titin fragments to enter the bloodstream and be excreted in urine via glomerular filtration.

Nonetheless, as shown in Fig. 1(a), UTF concentrations did not increase in all participants following exercise, indicating that — like CK — UTF exhibits considerable inter-individual variability. While differences in training status were controlled for by recruiting only strength-trained individuals, several biochemical mechanisms may explain this variability. Firstly, some individuals may have inherently more robust sarcomeric structures, potentially due to differential expression of titin isoforms. Specifically, a higher proportion of the more compliant N2BA isoform, as opposed to the stiffer N2B variant, could confer greater sarcomere elasticity, reducing mechanical strain and titin disruption under eccentric loading (Lewinter and Granzier 2010). Secondly, the proteolytic activity of calpains, which degrade titin, is highly Ca^2^⁺ dependent (Gissel 2005). Therefore, individual differences in calcium homeostasis—influenced by factors such as sarcoplasmic reticulum function, ion channel behaviour, and mitochondrial buffering capacity—could modulate calpain activation and, consequently, titin degradation. These mechanisms together may account for the non-responder phenotype observed in some participants and subsequently may be a limiting factor for using UTF as a biomarker for detecting EIMD.

EIMD induces a prolonged reduction in muscle force-generating capacity that can last for several days (Byrne et al. 2004). As such, changes in neuromuscular function are viewed as the most appropriate marker of EIMD (Warren et al. 1999; Paulsen et al. 2012; Damas et al. 2016). Hence, we measured an array of neuromuscular responses to determine whether the IKD exercise protocol induced EIMD, and whether changes in UTF reflected EIMD. In this study, we observed that exercise elicited changes in markers of neuromuscular function; however, after 48 h, only MVIF remained attenuated. A reduction in MVIF without concurrent impairments in VA, RFD_max_, or twitch potentiation does not necessarily indicate that EIMD was absent but rather suggests that the damage may have been relatively mild, predominantly mechanical rather than neural, and that recovery processes were likely already underway—particularly among more trained individuals. Additionally, there was considerable inter-individual variability in neuromuscular responses: while some participants returned to baseline by 48 h, others exhibited persistent deficits across multiple markers. It is also important to acknowledge that the sample size for MVIF (*n* = 27) was larger than that for VA, Tw_pot_, TTP, and RFD_max_ (*n* = 17), which may have increased the sensitivity to detect changes in MVIF relative to the other neuromuscular measures.

DOMS is another commonly reported manifestation of EIMD, typically appearing 12–48 h post-exercise and peaking between 24 and 72 h (Jones et al. 1989; Clarkson et al. 1992; Cleak and Eston 1992). Although widely used as an indirect marker of EIMD, DOMS exhibits a poor temporal relationship with histological evidence of ultrastructural muscle damage (Clarkson et al. 1992). Nonetheless, previous studies have reported positive correlations between UTF concentrations and DOMS following muscle-damaging exercise (Kanda et al. 2017; Yamaguchi et al. 2020c). In the present study, DOMS was significantly elevated at 48 h post-exercise, suggesting that EIMD may have occurred despite a lack of consistent responses in some neuromuscular markers. However, this increase in DOMS did not correlate with changes in UTF. Several factors may explain the discrepancy between our findings and earlier studies reporting a positive relationship between UTF and DOMS, including: (1) differences in the muscle groups targeted (quadriceps in the present study vs. gastrocnemius (Kanda et al. 2017) or elbow flexors (Yamaguchi et al. 2020c)) (2) participant sex, as previous studies included only males; (3) variation in the assessment tools used to quantify DOMS (e.g. 200 mm vs. 100 mm visual analogue scales); and (4) differences in participant training status, as those in the current study had prior resistance training experience, whereas the training backgrounds of participants in earlier studies were not reported (Kanda et al. 2017; Yamaguchi et al. 2020c).

Correlations were performed to determine whether UTF could serve as a valid intramuscular biomarker for detecting the functional manifestations of EIMD (DOMS and neuromuscular function). Given that neuromuscular function is considered the gold standard marker of EIMD (Warren et al. 1999), a strong correlation with these markers would suggest UTF could be an appropriate biomarker of EIMD, which is less invasive than the most commonly measured intramuscular protein, serum CK. Overall, we found that changes in UTF correlated more strongly with changes in neuromuscular function markers than changes in CK 48 h after exercise. Specifically, changes in UTF were negatively correlated with MVIF, Tw_pot_ and evoked RFD_max_, whilst changes in CK correlated with DOMS and evoked RFD_max_. Although this is the first study to examine and establish an association between UTF and TW_pot_ and evoked RFD_max_, the temporal relationship between UTF and MVIF has been reported elsewhere; Yamaguchi et al. (2020c) similarly found a strong negative correlation between these markers from 48 to 144 h post-EIMD, whilst Tanabe et al. (2021b) reported a moderate negative correlation between peak MVIF and peak UTF. This close association is likely explained by titin’s key role in generating passive force in skeletal muscle and therefore overall force production (Freundt and Linke 2019). As reported previously (Yamaguchi et al. 2020c), UTF was also strongly correlated with CK. Collectively, our findings add to the growing literature that suggests UTF is a potentially useful biomarker of myofibrillar damage following strenuous exercise. Given the strong correlation between UTF and CK at 48 h post-exercise, UTF could be the preferred marker for the indirect detection of damage to intramuscular proteins.

This is the first study to examine exercise-induced UTF levels in females. We hypothesised that females would have lower UTF responses to exercise given that some studies found that females (from human and rodent models) experience less severe symptoms of EIMD after eccentric exercise compared to males (Bär et al. 1988; Amelink et al. 1988; Dernbach et al. 1993; St Pierre Schneider et al. 1999; Carter et al. 2001). However, evidence remains inconsistent (Clarkson and Hubal 2001). The rationale for a blunted EIMD response in females is attributed to various factors, notably increased levels of oestrogen, which may have a protective effect on muscle damage by modulating inflammation (Carter et al. 2001). Furthermore, males have a higher proportion of type II muscle fibres, which are more susceptible to damage from eccentric exercise (Dannecker et al. 2012). However, this study suggests that exercise-induced UTF concentrations are not different between males and females. Other than the small differences in TTP at 48 h post-exercise between males and females, there was no evidence of sex differences in markers of EIMD. Thus, our findings suggest that UTF could be used as a marker of EIMD in both males and females.

This study has several limitations that should be acknowledged. Firstly, we did not include a concentric-biased, non-damaging exercise control group which would have enabled us to confirm whether increases in UTF were specifically attributable to EIMD rather than other mechano-physiological responses to exercise. However, Yamaguchi et al. (2020a) have previously demonstrated that UTF levels increase to a greater extent following eccentric compared to concentric exercise, thereby supporting the link between UTF elevation and EIMD. Secondly, measurements were only taken at 48 h post-exercise due to limited resources which limited our ability to replicate the findings by Kanda et al. (2017) that highlighted UTF levels tended to peak at 96 h post-exercise. This meant that we missed an opportunity to assess temporal dynamics, including potential sex differences or stronger correlations, which may have emerged at later time points. Additionally, the data presented here are part of an exploratory analysis within a larger study, and an a priori power calculation was not conducted specifically for this sub-analysis. As such, the study may have been underpowered to detect smaller effects or interactions. Notwithstanding these limitations, this study had a larger sample size than all previous investigations examining UTF responses to exercise and, importantly, was the first to characterise UTF responses in females. Furthermore, the inclusion of a comprehensive range of neuromuscular function markers, which have not previously been evaluated alongside UTF, strengthens the novelty and translational relevance of our findings. Together, these data provide new insights into the potential utility of UTF as a biomarker of EIMD in physically active males and females.

## Conclusion

In conclusion, strenuous, eccentric-heavy exercise significantly increased UTF concentrations in some individuals 48 h post-exercise. Notably, this study is the first to demonstrate that UTF increases similarly in both males and females. Compared to CK, UTF showed a stronger association with exercise-induced deficits in neuromuscular function, although not with DOMS. However, similar to CK, UTF responses exhibited substantial inter-individual variability. These findings suggest that while UTF may better reflect the extent of EIMD than CK, its variable responsiveness currently limits its reliability as a standalone biomarker.

## Supplementary Information

Below is the link to the electronic supplementary material.

Supplementary file1 (DOCX 16 KB)

## Data Availability

The datasets used and/or analysed during the current study are available from the corresponding author on reasonable request.

## References

[CR1] Ali MAM, Cho WJ, Hudson B et al (2010) Titin is a target of matrix metalloproteinase-2: Implications in myocardial ischemia/reperfusion injury. Circulation 122:2039–2047. 10.1161/CIRCULATIONAHA.109.930222/SUPPL_FILE/CIR201009-ONLINE_SUPPLEMENTARY.PDF 21041693 10.1161/CIRCULATIONAHA.109.930222 PMC3057897

[CR2] Amelink GJ, Kamp HH, Bär PR (1988) Creatine kinase isoenzyme profiles after exercise in the rat: sex-linked differences in leakage of CK-MM. Pflugers Arch 412:417–421. 10.1007/BF01907561 3174399 10.1007/BF01907561

[CR3] Bär PR, Amelink GJ, Oldenburg B, Blankenstein MA (1988) Prevention of exercise-induced muscle membrane damage by oestradiol. Life Sci 42:2677–2681. 10.1016/0024-3205(88)90243-3 3386407 10.1016/0024-3205(88)90243-3

[CR4] Beckmann JS, Spencer M (2008) Calpain 3, the “gatekeeper” of proper sarcomere assembly, turnover and maintenance. Neuromuscul Disord 18:913–921. 10.1016/J.NMD.2008.08.005 18974005 10.1016/j.nmd.2008.08.005 PMC2614824

[CR5] Behrens M, Mau-Moeller A, Bruhn S (2012) Effect of exercise-induced muscle damage on neuromuscular function of the quadriceps muscle. Int J Sports Med 33:600–606. 10.1055/S-0032-1304642 22510801 10.1055/s-0032-1304642

[CR6] Brancaccio P, Maffulli N, Limongelli FM (2007) Creatine kinase monitoring in sport medicine. Br Med Bull 81–82:209–230. 10.1093/BMB/LDM014 17569697 10.1093/bmb/ldm014

[CR7] Brancaccio P, Lippi G, Maffulli N (2010) Biochemical markers of muscular damage. Clin Chem Lab Med 48:757–767. 10.1515/CCLM.2010.179 20518645 10.1515/CCLM.2010.179

[CR8] Byrne C, Twist C, Eston R (2004) Neuromuscular function after exercise-induced muscle damage: theoretical and applied implications. Sports Med 34:49–69. 10.2165/00007256-200434010-00005 14715039 10.2165/00007256-200434010-00005

[CR9] Carter A, Dobridge J, Hackney AC (2001) Influence of estrogen on markers of muscle tissue damage following eccentric exercise. Human Physiol 27(5):626–630. 10.1023/A:1012395831685 11680291

[CR10] Cermak NM, Noseworthy MD, Bourgeois JM et al (2012) Diffusion tensor MRI to assess skeletal muscle disruption following eccentric exercise. Muscle Nerve 46:42–50. 10.1002/MUS.23276 22644795 10.1002/mus.23276

[CR11] Charton K, Sarparanta J, Milic A et al (2015) Complex relationship between calpain 3 and titin. Neuromuscul Disord 25:S232. 10.1016/j.nmd.2015.06.171

[CR12] Clarkson PM, Hubal MJ (2001) Are women less susceptible to exercise-induced muscle damage? Curr Opin Clin Nutr Metab Care 4:527–531. 10.1097/00075197-200111000-00011 11706288 10.1097/00075197-200111000-00011

[CR13] Clarkson PM, Hubal MJ (2002) Exercise-induced muscle damage in humans. Am J Phys Med Rehabil 81:S52-69. 10.1097/00002060-200211001-00007 12409811 10.1097/00002060-200211001-00007

[CR14] Clarkson PM, Nosaka K, Braun B (1992) Muscle function after exercise-induced muscle damage and rapid adaptation. Med Sci Sports Exerc 24:512–520. 10.1249/00005768-199205000-00004 1569847

[CR15] Cleak MJ, Eston RG (1992) Muscle soreness, swelling, stiffness and strength loss after intense eccentric exercise. Br J Sports Med 26:267–272. 10.1136/BJSM.26.4.267 1490222 10.1136/bjsm.26.4.267 PMC1479005

[CR16] Cohen J (1988) Statistical Power Analysis for the Social Sciences, 2nd edn. Lawrence Erlbaum Associates, Hillsdale, New Jersey

[CR17] Damas F, Nosaka K, Libardi CA et al (2016) Susceptibility to exercise-induced muscle damage: a cluster analysis with a large sample. Int J Sports Med 37:633–640. 10.1055/S-0042-100281 27116346 10.1055/s-0042-100281

[CR18] Dancey CP, Reidy J (2007) Statistics Without Maths for Psychology. Pearson Education

[CR19] Dannecker EA, Liu Y, Rector RS et al (2012) Sex differences in exercise-induced muscle pain and muscle damage. J Pain: off J Am Pain Soc 13:1242. 10.1016/J.JPAIN.2012.09.014 10.1016/j.jpain.2012.09.014 PMC3513404 23182229

[CR20] Dernbach AR, Sherman WM, Simonsen JC et al (1993) No evidence of oxidant stress during high-intensity rowing training. J Appl Physiol 74:2140–2145. 10.1152/JAPPL.1993.74.5.2140 8335541 10.1152/jappl.1993.74.5.2140

[CR21] Deyhle MR, Sorensen JR, Hyldahl RD (2016) Induction and Assessment of Exertional Skeletal Muscle Damage in Humans. J vis Exp 2016:54859. 10.3791/54859 10.3791/54859 PMC5226389 28060273

[CR22] Doguet V, Jubeau M, Dorel S et al (2016) Time-course of neuromuscular changes during and after maximal eccentric contractions. Front Physiol 7:196948. 10.3389/FPHYS.2016.00137 10.3389/fphys.2016.00137 PMC4834740 27148075

[CR23] Farup J, Rahbek SK, Bjerre J et al (2016) Associated decrements in rate of force development and neural drive after maximal eccentric exercise. Scand J Med Sci Sports 26:498–506. 10.1111/SMS.12481 25944178 10.1111/sms.12481

[CR24] Fatouros IG, Jamurtas AZ (2016) Insights into the molecular etiology of exercise-induced inflammation: opportunities for optimizing performance. J Inflamm Res 9:175–186. 10.2147/JIR.S114635 27799809 10.2147/JIR.S114635 PMC5085309

[CR25] Freundt JK, Linke WA (2019) Titin as a force-generating muscle protein under regulatory control. J Appl Physiol 126:1474–1482. 10.1152/JAPPLPHYSIOL.00865.2018 30521425 10.1152/japplphysiol.00865.2018

[CR26] Friden J, Sjostrom M, Ekblom B (1983) Myofibrillar damage following intense eccentric exercise in man. Int J Sports Med 4:170–176. 10.1055/S-2008-1026030 6629599 10.1055/s-2008-1026030

[CR27] Fridén J, Lieber RL (2001) Eccentric exercise-induced injuries to contractile and cytoskeletal muscle fibre components. Acta Physiol Scand 171:321–326. 10.1046/J.1365-201X.2001.00834.X 11412144 10.1046/j.1365-201x.2001.00834.x

[CR28] Gissel H (2005) The role of Ca2+ in muscle cell damage. Ann N Y Acad Sci 1066:166–180. 10.1196/ANNALS.1363.013 16533926 10.1196/annals.1363.013

[CR29] Herzog W (2018) The multiple roles of titin in muscle contraction and force production. Biophys Rev 10:1187–1199. 10.1007/S12551-017-0395-Y 29353351 10.1007/s12551-017-0395-y PMC6082311

[CR30] Howatson G, Van Someren KA (2008) The prevention and treatment of exercise-induced muscle damage. Sports Med. 10.2165/00007256-200838060-00004 18489195 10.2165/00007256-200838060-00004

[CR31] Hyldahl RD, Hubal MJ (2014) Lengthening our perspective: morphological, cellular, and molecular responses to eccentric exercise. Muscle Nerve 49:155–170. 10.1002/MUS.24077 24030935 10.1002/mus.24077

[CR32] Ishii MN, Nakashima M, Kamiguchi H et al (2023) Urine titin as a novel biomarker for Duchenne muscular dystrophy. Neuromuscul Disord 33:302–308. 10.1016/j.nmd.2023.02.003 36871413 10.1016/j.nmd.2023.02.003

[CR33] Janecki D, Jaskólska A, Marusiak J et al (2014) Twitch mechanical properties after repeated eccentric exercise of the elbow flexors. Appl Physiol Nutr Metab 39:74–81. 10.1139/APNM-2013-0097 24383510 10.1139/apnm-2013-0097

[CR34] Jenkins NDM, Housh TJ, Traylor DA et al (2014) The rate of torque development: a unique, non-invasive indicator of eccentric-induced muscle damage? Int J Sports Med 35:1190–1195. 10.1055/S-0034-1375696 25259592 10.1055/s-0034-1375696

[CR35] Jones DA, Newham DJ, Torgan C (1989) Mechanical influences on long-lasting human muscle fatigue and delayed-onset pain. J Physiol 412:415. 10.1113/JPHYSIOL.1989.SP017624 2600839 10.1113/jphysiol.1989.sp017624 PMC1190584

[CR36] Kanda K, Sakuma J, Akimoto T et al (2017) Detection of titin fragments in urine in response to exercise-induced muscle damage. PLoS ONE 12:e0181623. 10.1371/JOURNAL.PONE.0181623 28727760 10.1371/journal.pone.0181623 PMC5519174

[CR37] Kendall B, Eston R (2002) Exercise-induced muscle damage and the potential protective role of estrogen. Sports Med 32:103–123. 10.2165/00007256-200232020-00003/FIGURES/2 11817996 10.2165/00007256-200232020-00003

[CR38] Letavernier E, Zafrani L, Perez J et al (2012) The role of calpains in myocardial remodelling and heart failure. Cardiovasc Res 96:38–45. 10.1093/CVR/CVS099 22425901 10.1093/cvr/cvs099

[CR39] Lewinter MM, Granzier H (2010) Cardiac Titin - a multifunctional giant: lewinter and granzier: cardiac titin. Circulation 121:2137. 10.1161/CIRCULATIONAHA.109.860171 20479164 10.1161/CIRCULATIONAHA.109.860171 PMC2905226

[CR40] Margaritis I, Tessier F, Verdera F et al (1999) Muscle enzyme release does not predict muscle function impairment after triathlon. J Sports Med Phys Fitness 39:133–139 10399422

[CR41] Maruyama N, Asai T, Abe C et al (2016) Establishment of a highly sensitive sandwich ELISA for the N-terminal fragment of titin in urine. Sci Rep 6(1):1–11. 10.1038/srep39375 27991570 10.1038/srep39375 PMC5171804

[CR42] Matsuo M, Awano H, Maruyama N, Nishio H (2019) Titin fragment in urine: a noninvasive biomarker of muscle degradation. Adv Clin Chem 90:1–23. 10.1016/BS.ACC.2019.01.001 31122607 10.1016/bs.acc.2019.01.001

[CR43] Millet GY, Lepers R (2004) Alterations of Neuromuscular Function after Prolonged Running, Cycling and Skiing Exercises. Sports Med 34:105–116. 10.2165/00007256-200434020-00004 14965189 10.2165/00007256-200434020-00004

[CR44] Molina R, Denadai BS (2012) Dissociated time course recovery between rate of force development and peak torque after eccentric exercise. Clin Physiol Funct Imaging 32:179–184. 10.1111/J.1475-097X.2011.01074.X 22487151 10.1111/j.1475-097X.2011.01074.x

[CR45] Nakanishi N, Tsutsumi R, Hara K et al (2021) Urinary titin n-fragment as a biomarker of muscle atrophy, intensive care unit-acquired weakness, and possible application for post-intensive care syndrome. J Clin Med 10:1–4. 10.3390/JCM10040614 10.3390/jcm10040614 PMC7915692 33561946

[CR46] Nosaka K, Clarkson PM (1996) Variability in serum creatine kinase response after eccentric exercise of the elbow flexors. Int J Sports Med 17:120–127. 10.1055/S-2007-972819 8833714 10.1055/s-2007-972819

[CR47] Paulsen G, Mikkelsen UR, Raastad T, Peake JM (2012) Leucocytes, cytokines and satellite cells: what role do they play in muscle damage and regeneration following eccentric exercise? Exerc Immunol Rev 18:42–97 22876722

[CR48] Penailillo L, Gurovich A, Plaza P, Nosaka K (2011) Sex differences in the changes in muscle damage markers following eccentric exercise of the elbow flexors. J Sci Med Sport 14:e100–e101. 10.1016/j.jsams.2011.11.210

[CR49] Power GA, Dalton BH, Rice CL, Vandervoort AA (2010) Delayed recovery of velocity-dependent power loss following eccentric actions of the ankle dorsiflexors. J Appl Physiol 109:669–676. 10.1152/JAPPLPHYSIOL.01254.2009 20576845 10.1152/japplphysiol.01254.2009 PMC2944637

[CR50] Prado LG, Makarenko I, Andresen C et al (2005) Isoform diversity of giant proteins in relation to passive and active contractile properties of rabbit skeletal muscles. J Gen Physiol 126:461. 10.1085/JGP.200509364 16230467 10.1085/jgp.200509364 PMC2266601

[CR51] Prasartwuth O, Taylor JL, Gandevia SC (2005) Maximal force, voluntary activation and muscle soreness after eccentric damage to human elbow flexor muscles. J Physiol 567:337. 10.1113/JPHYSIOL.2005.087767 15946963 10.1113/jphysiol.2005.087767 PMC1474152

[CR52] Seifert JG, Kipp RW, Amann M, Gazal O (2005) Muscle damage, fluid ingestion, and energy supplementation during recreational alpine skiing. Int J Sport Nutr Exerc Metab 15:528–536. 10.1123/IJSNEM.15.5.528 16327033 10.1123/ijsnem.15.5.528

[CR53] St Pierre Schneider B, Correia LA, Cannon JG (1999) Sex differences in leukocyte invasion in injured murine skeletal muscle. Res Nurs Health 22:243–251. 10.1002/(SICI)1098-240X(199906)22:3 10344704 10.1002/(sici)1098-240x(199906)22:3<243::aid-nur6>3.0.co;2-x

[CR54] Sun J, Ye S, Yin G, Xie Q (2023) The diagnostic value of urinary N-terminal fragment of titin for skeletal muscle damage in idiopathic inflammatory myopathy. Rheumatology (Oxford) 62:3742–3748. 10.1093/RHEUMATOLOGY/KEAD109 36919777 10.1093/rheumatology/kead109

[CR55] Tanabe Y, Shimizu K, Kondo E et al (2021a) Urinary n-terminal fragment of Titin reflects muscle damage after a soccer match in male collegiate soccer players. J Strength Cond Res 35:360–365. 10.1519/JSC.0000000000003923 33337691 10.1519/JSC.0000000000003923

[CR56] Tanabe Y, Shimizu K, Sagayama H et al (2021b) Urinary N-terminal fragment of titin: a surrogate marker of serum creatine kinase activity after exercise-induced severe muscle damage. J Sports Sci 39:1437–1444. 10.1080/02640414.2021.1876329 33722155 10.1080/02640414.2021.1876329

[CR57] Trappe TA, Carrithers JA, White F et al (2002) Titin and nebulin content in human skeletal muscle following eccentric resistance exercise. Muscle Nerve 25:289–292. 10.1002/mus.10037 11870701 10.1002/mus.10037

[CR58] Turner TS, Tucker KJ, Rogasch NC, Semmler JG (2008) Impaired neuromuscular function during isometric, shortening, and lengthening contractions after exercise-induced damage to elbow flexor muscles. J Appl Physiol 105:502–509. 10.1152/JAPPLPHYSIOL.90421.2008 18556432 10.1152/japplphysiol.90421.2008

[CR59] Vila-Chã C, Bovolini A, Francisco C et al (2023) Acute effects of isotonic eccentric exercise on the neuromuscular function of knee extensors vary according to the motor task: impact on muscle strength profiles, proprioception and balance. Front Sports Act Living 5:1273152. 10.3389/FSPOR.2023.1273152 38022776 10.3389/fspor.2023.1273152 PMC10655025

[CR60] Warren GL, Lowe DA, Armstrong RB (1999) Measurement tools used in the study of eccentric contraction-induced injury. Sports Med 27:43–59. 10.2165/00007256-199927010-00004 10028132 10.2165/00007256-199927010-00004

[CR61] Yamaguchi S, Suzuki K, Inami T et al (2020a) Changes in urinary titin N-terminal fragment concentration after concentric and eccentric exercise. J Sports Sci Med 19:121–129 32132835 PMC7039023

[CR62] Yamaguchi S, Suzuki K, Kanda K et al (2020b) Changes in urinary titin N-terminal fragments as a biomarker of exercise-induced muscle damage in the repeated bout effect. J Sci Med Sport 23:536–540. 10.1016/J.JSAMS.2019.12.023 31928880 10.1016/j.jsams.2019.12.023

[CR63] Yamaguchi S, Suzuki K, Kanda K, Okada J (2020c) N-terminal fragments of titin in urine as a biomarker for eccentric exercise-induced muscle damage. J Phys Fit Sports Med 9:21–29. 10.7600/JPFSM.9.21

