# Supplementary material for: Urinary N-terminal titin fragment concentration as a non-invasive biomarker of exercise-induced muscle damage in males and females
Source: Eur J Appl Physiol. 2025 Aug 12;126(2):787–99. doi: 10.1007/s00421-025-05936-6 (PMC12948870; doi:10.1007/s00421-025-05936-6)
Supplement: Supplementary file 1 — Supplementary file1 (DOCX 16 KB) [file 421_2025_5936_MOESM1_ESM.docx]

**Supplementary Table 1:** Absolute values for changes in neuromuscular function and muscle soreness after strenuous exercise.

|  | **Pre-exercise** | | | **Post-exercise** | | | **48 hrs post-exercise** | | |
| --- | --- | --- | --- | --- | --- | --- | --- | --- | --- |
|  | Males | Females | **Overall** | Males | Females | **Overall** | Males | Females | **Overall** |
| **MVIF (N)** | 834±199 | 564±103 | **733±210** | 610±184 | 398±116 | **536±191** | 705±195 | 454±129 | **615±209** |
| **VA (%)** | 96±1 | 98±1 | **97±2** | 88±7 | 88±9 | **88±8** | 94 ±3 | 95±5 | **94±4** |
| **Tw_pot_ (N)** | 428±103 | 295±54 | **370±102** | 212±127 | 129±29 | **176±103** | 394±124 | 276±64 | **340±112** |
| **RFD_max_ (N/s)** | 4356±1047 | 3266±518 | **3859±976** | 2673±1508 | 1633±408 | **2208±1225** | 4057±1124 | 2907±584 | **3555±1026** |
| **TTP (ms)** | 77±14 | 60±14 | **71±15** | 55±10 | 58±9 | **57±10** | 71±6 | 76±9 | **73±8** |
| **DOMS (mm)** | 13±16 | 9±14 | **11±15** | 77±38 | 58±46 | **69±42** | 89±38 | 69±41 | **81±39** |
| Maximal voluntary isometric force (MVIF), voluntary activation (VA), potentiated twitch force (Tw_pot_), evoked maximal rate of force development (RFD_max_), time to peak twitch (TTP), delayed onset muscle soreness (DOMS). Values presented as mean ± SD. | | | | | | | | | |
